# Diversity and Control of Spoilage Fungi in Dairy Products: An Update

**DOI:** 10.3390/microorganisms5030042

**Published:** 2017-07-28

**Authors:** Lucille Garnier, Florence Valence, Jérôme Mounier

**Affiliations:** 1 Laboratoire Universitaire de Biodiversité et Ecologie Microbienne (LUBEM EA3882), Université de Brest, Technopole Brest-Iroise, 29280 Plouzané, France; lucille.garnier@univ-brest.fr; 2 Science et Technologie du Lait et de l’Œuf (STLO), AgroCampus Ouest, INRA, 35000 Rennes, France; florence.valence-bertel@inra.fr

**Keywords:** spoilage, fungi, dairy products, control, diversity

## Abstract

Fungi are common contaminants of dairy products, which provide a favorable niche for their growth. They are responsible for visible or non-visible defects, such as off-odor and -flavor, and lead to significant food waste and losses as well as important economic losses. Control of fungal spoilage is a major concern for industrials and scientists that are looking for efficient solutions to prevent and/or limit fungal spoilage in dairy products. Several traditional methods also called traditional hurdle technologies are implemented and combined to prevent and control such contaminations. Prevention methods include good manufacturing and hygiene practices, air filtration, and decontamination systems, while control methods include inactivation treatments, temperature control, and modified atmosphere packaging. However, despite technology advances in existing preservation methods, fungal spoilage is still an issue for dairy manufacturers and in recent years, new (bio) preservation technologies are being developed such as the use of bioprotective cultures. This review summarizes our current knowledge on the diversity of spoilage fungi in dairy products and the traditional and (potentially) new hurdle technologies to control their occurrence in dairy foods.

## 1. Introduction

Since people began producing and storing food products, spoilage and food losses and waste became important issues for human with regards to food safety and security. Nowadays, up to one third of all food is spoiled or squandered before consumption, which represents about 1.3 billion tons per year [1]. These losses are the results of one or more problems occurring in the supply chain, from initial agricultural production down to the consumer level [1]. Concerning food spoilage, a food product can be physically, chemically, or microbiologically spoiled. Parasites, bacteria, and/or fungi are the main agents causing microbial spoilage. Certain parasites and bacteria are of public health concern because they are common contaminants of foods and are often responsible for food outbreaks. Nonetheless, being ubiquitous in nature, fungi are also very common in food. For a long time, besides altering food properties, they were not regarded as particularly detrimental to human health, although *Claviceps purpurea* has been related to ergotism outbreaks in the Middle Ages more than 200 years ago [2]. It is only in recent times that several mycotoxins produced by certain fungal species have been regarded as a major threat to human and animal health, especially in developing countries, being responsible for different adverse health effects [3].

Yeasts and molds are able to grow in a large variety of food including raw materials such as cereals, vegetables, fruits, meat, and milk, as well as processed products [4]. Given this large variety, fungal spoilage leads to severe economic losses for food manufacturers. Indeed, it is estimated that between 5% and 10% of the world’s food production is lost due to fungal deterioration [2]. Dairy products are less susceptible to fungal spoilage than other products, such as fruits or vegetables, because they are usually kept at refrigerated temperatures, are often made with heat-treated milk, and some of them are fermented products meaning that they may possess a competitive microbiota, have an acidic pH, and naturally contain organic acids. Nonetheless, a significant number of fungal species are able to survive and grow in dairy products. This astonishing adaptation capacity may be explained by the ability of fungi to utilize numerous substrates including carbohydrates, organic acids, proteins, and lipids that are present in milk and its derived products [5]. Moreover, these fungi are acidotolerant, xerotolerant, and/or psychrotolerant, and to some extent can tolerate chemical preservatives, which are sometimes added to increase product shelf-life. It is worth mentioning that several fungal species such as *Debaryomyces hansenii*, *Candida catenulata*, *Galactomyces geotrichum*, *Kluyveromyces marxianus*, *Mucor lanceolatus*, *Penicillium roqueforti*, *Penicillium camemberti*, or *Saccharomyces cerevisiae* are deliberately added as technological adjunct cultures to manufacture dairy products including kefir-type products and many kinds of cheese varieties [6,7,8]. Concerning undesirable species, their presence in dairy products may result in several types of food spoilage, e.g., visible growth of the fungus at the product surface, and the production of metabolites causing off-odors and flavors, as well as visible changes in color and/or texture [9]. In addition to organoleptic properties’ deterioration, spoilage molds such as *Penicillium* and *Aspergillus* spp. can also produce mycotoxins [4,5,10,11]. In milk and dairy products, aflatoxin M1 (AFM1), which is produced by certain *Aspergillus* species, is the only mycotoxin for which maximum levels (0.05 ppb in the European Union (EU)) have been established. The occurrence of AFM1 in milk results from the conversion by dairy animals, fed on aflatoxin B1 (AFB1)-contaminated feedstuffs, of AFB1 to AFM1, which then pass to their urine and milk. Other mycotoxins such as ochratoxin A, citrinin, roquefortin C, mycophenolic, and cyclopiazonic acids have also been detected in cheeses at various concentrations [10]. Except for AFM1, intake of mycotoxins from dairy products is generally considered of limited importance compared to cereals and their derived products, and no human case of food poisoning related to mycotoxins has been documented so far.

Control of fungal spoilage is a major concern for industrials and scientists that are looking for efficient solutions to prevent and/or limit fungal growth or development in dairy products. Different traditional methods, also called traditional hurdle technologies, are implemented to control such contaminations including air treatment, cleaning and disinfection procedures, heat treatment, water activity reduction by brining, refrigeration, modified atmosphere packaging [12], as well as the use of chemical preservatives which are considered as food additives. The spoilage frequency and rate of many dairy foods can be reduced or retarded by the application of one or more of these treatments. However, fungal spoilage is still an issue for dairy manufacturers. Indeed, increasing fungal resistance toward heat treatments or chemical preservatives [13,14] and consumers’ demand for more “natural” products, as well as legislation evolution have led industrial dairy producers to find complementary control approaches. This situation has led to the development of new (bio) preservation technologies such as the use of bioprotective cultures [15].

This review summarizes our current knowledge on spoilage fungi in dairy products with a special emphasis on their diversity, as well as the traditional and (potentially) new hurdle technologies to control their occurrence in dairy products. 

## 2. Diversity of Spoilage Fungi in Dairy Products

With the use of multilocus sequencing of DNA regions with taxonomical interest, fungal taxonomy has undergone important changes during the last fifteen years. Many species have been reclassified and new phylogenetic species are also being regularly recognized within so-called “species complexes,” in which members only harbor few differences in their morphological characters or are even morphologically undistinguishable from each other. For fungal identification, the internal transcribed spacer region (ITS) has been chosen as the best universal barcode [16]. Concerning yeast identification, the D1/D2 domain of the 28 S rRNA gene is also widely used. It is worth mentioning that these genes are not always sufficient to identify isolates at the species level, and sequencing of other genes can be required. This is especially true for members of the *Aspergillus*, *Cladosporium*, *Penicillium*, and *Phoma* genera. 

Over the years, the microbial diversity of milk and fermented dairy products has received considerable attention, and the development of next-generation sequencing (NGS) technologies [17,18,19,20,21,22] offers novel and rapid methods to characterize food ecosystems. However, except for raw milk, these techniques are not widely used to investigate fungal spoilage of dairy products because spoilage is generally the consequence of the outgrowth of a few species at the same time and because contamination can also occur on a distinct spot of the product surface. In addition, because read length is still limited with the currently used NGS technologies and because the taxonomic resolution of a single barcode marker can vary among taxa, it can be very tricky to identify operational taxonomic units (OTUs) at the species level. Nevertheless, these techniques offer new possibilities to investigate the potential sources of fungi in the dairy environment, which constitutes an important source of spoilage microorganisms.

### 2.1. Sources of Fungal Contamination

Fungal contamination of dairy foods can occur at different stages, from dairy farms to dairy processing units and at consumers’ homes. Independent of the animal species, raw milk generally contains between 3 to 5 log 10 CFU·mL^−1^ fungi with higher number of yeast cells than fungal spores [23,24]. As shown by a recent study [25], the stable and milking parlor environments at the farm are important sources of fungi in the milk. In addition, an important yeast source is the teat surface [25]. It should be mentioned that yeast growth during milk storage is rare, as yeasts are rapidly outnumbered by psychrotrophic bacteria such as *Pseudomonas* spp. [26,27]. Moreover, except for a few fungal species, yeast and molds are not heat-resistant and should be killed after pasteurization. Therefore, during manufacturing, fungal contamination generally occurs after milk heat-treatment. Mold spoilage is often due to airborne fungi because fungal spores are easily dispersed into the dairy plant air [28]. In a recent study undertaken in a Greek dairy plant, fungal counts of 362.3 CFU/m^3^ consisting mainly of mold spores were reported in outdoor air samples while 69.8 CFU/m^3^ and 266.2 CFU/m^3^ wer found in samples from two indoor locations [29]. Recent studies [28,29,30,31,32,33] investigated the fungal diversity of the dairy environment in different dairy plants, and mold contaminations were also shown to originate from the air. In the case of sliced cheese, the cheese rind itself can also be a source of spoilage fungi which are transferred during cheese slicing to the cheese slice surface and will grow during storage at the retailer or at the consumer’s home [30]. In contrast, yeast causing spoilage generally originates from brine, surface, equipment, or ingredient contaminations [32,34,35,36], but can also be detected in the air [29]. The brine used for cheese salting/curing is one of the most significant sources of fungi. In one study [32], counts of 1.10^9^ CFU/cm^2^ were reported in a brining tank. Moreover, ingredients such as fruit preparations can also be important contamination sources for both yeast and molds [4,37]. In addition, certain fruit preparations such as blueberries and strawberries are even more at risk as they do not support extensive heat-treatment [38]. It is also worth mentioning that 50–100% fruit preparations including lemon cells, strawberries, and blueberries were recently reported to contain heat-resistant ascospore-forming fungi [39]. Finally, packaging materials may also be a source of spoilage molds, despite the fact that it has not been extensively studied.

Athough there has been limited research on the subject, fungal contaminations are also likely to occur at the consumer’s home after product opening. In a recent study on 586 surface samples collected from ~293 refrigerators in Italy [40], 15% and 5% of total samples contained fungal populations >1 log10 CFU·cm^−2^ and >2.5 log10 CFU·cm^−2^, respectively. Besides refrigerator surfaces, cross-contamination from one product to another can also be responsible for fungal contamination. For example, mold- and smear-ripened cheeses as well as vegetables and fruits are potential yeast and mold contamination sources. Finally, the indoor air at a consumer’s home is also a potent source of spoilage molds. 

### 2.2. Spoilage Yeasts

Dairy food spoilage caused by yeast can result in visible alterations mainly due to their outgrowth on the product surface, such as the “toad skin” defect caused by *G. geotrichum* or browning reaction caused by *Yarrowia lipolytica*. The latter defect is due to the extracellular accumulation of homogentisic acid, an intermediate of tyrosine catabolism, capable of auto-oxidization and polymerization, leading to the formation of pyomelanin, a brown pigment [41,42]. Yeast spoilage may also lead to detectable but non-visible alterations resulting in off-odor and -flavor or texture alterations through the production of ethanol, CO_2_, and volatile organic compounds (primary and secondary alcohols, aldehydes, esters) as well as the production of lipolytic and proteolytic enzymes (glycolysis). One should keep in mind that the extent of spoilage depends on two parameters: the minimal spoilage level and the chemical spoilage index, which represent the concentrations of specific spoilage organism(s) and that of spoilage metabolites determined at the time of sensory rejection, respectively [43]. In relation to yeast spoilage in dairy products, little data are available on these values. It was defined that yeasty and fermented off-flavors were detected when yeasts grew at populations equal or above 10^5^–10^6^ CFU/g [9]. 

It is also worth mentioning that certain yeasts species are able to produce biogenic amines (BA) including histamine and tyramine [44,45,46]. For example, *Y. lipolytica* was responsible for histidine, lysine, ornithine, phenylalanine, and tyrosine decarboxylation, leading to the production of putrescine, 2-phenylethylamine, tyramine, and cadaverine, respectively, in a traditional Italian cheese [44]. However, the main BA producers in dairy products are bacteria including lactic acid bacteria (LAB) and enterobacteria, particularly with the formation of histamine and tyramine [47]. Interestingly, it has already been shown that *G. geotrichum* was able to slightly degrade BA such as tyramine [48]. 

A significant number of studies have been performed to assess spoilage yeast diversity (Table 1). These contaminations can be due to one or several species. For example, a recent study showed that up to 14 different yeast species could be encountered in as low as six fresh cheese samples, including cases of multi-contaminations [49]. Until now, more than 60 yeast species have been identified as spoilage agents of dairy products (Table 1). They belong to the Ascomycota and Basidiomycota phylum represented by 20 and 10 genera, respectively. Among Ascomycota, all identified species belong to the Saccharomycotina subdivision and Saccharomycetes class, which is the only class of this subdivision. Among this class, the genus *Candida* is the most frequently reported, representing half of the Ascomycota diversity, and is also characterized by a high diversity at the species level. Indeed, 24 different *Candida* species have already been reported as responsible for dairy product spoilage. *Candida parapsilosis* is the most frequently isolated species, followed by *Candida lusitaniae*, *Candida inconspicua*, and *Candida intermedia*. All these species can thus be considered common contaminants of dairy products. Table 1 also shows that dairy products spoiled by *Candida* are principally unripened products and hard or semi-hard cheeses such as Scamorza or Caciotta. 

Besides *Candida* spp., *Debaryomyces*, *Kluyveromyces*, *Yarrowia*, *Galactomyces*, and *Saccharomyces* spp. are also frequent spoilers of fresh dairy products (fresh cheese, cream, and yoghurt) and heat-treated products (Table 1). The presence of spoilage yeasts in the latter products is principally due to post-heat-treatment contaminations, but certain yeast species show high resistance to pasteurization [50] (see Section 3.2.1). Contrary to the genus *Candida*, one or two species from each genus are considered dairy spoilage agents. Indeed, the *Kluyveromyces* genus is represented by *Kluyveromyces marxianus* and *Kluyveromyces lactis. Debaryomyces*, *Galactomyces*, *Saccharomyces*, and *Yarrowia* genera are represented by *D. hansenii*, *G. geotrichum*, *S. cerevisiae*, and *Y. lipolytica*, respectively. Other Ascomycota yeasts responsible for spoilage include *Meyerozyma* (formerly *Pichia*) and *Pichia*, *Geotrichum*, *Dekkera*, *Torulaspora*, *Wickerhamomyces*, *Blastodendrion*, *Cyberlindnera*, *Kazachstania*, *Peterozyma*, *Priceomyces*, and *Torulopsis* spp., principally isolated from soft and unripened dairy products such as fresh cheese or yoghurt.

Among Basidiomycota, six genera and four genera belonging to the Agaromycotina and Pucciniomycotina subdivisions are regularly encountered in spoiled dairy products. Among Pucciniomycotina, the genus *Rhodotorula* (Microbotryomycetes class), represented by three different species, i.e., *Rhodotorula diffluens*, *Rhodotorula glutinis*, and *Rhodotorula mucilaginosa*, is the most frequently cited and is essentially isolated from unripened products (Table 1). The latter genus is followed by *Cryptococcus* (Tremellomycetes class), represented by four species, i.e., *Cryptococcus humicola*, *Cryptococcus laurentii*, *Cryptococcus pseudolongus*, and *Cryptococcus minuta* isolated from unripened products (fresh cheese and butter/margarine) and soft cheese (Table 1). Other *Basidiomycota* genera include *Saitozyma*, *Naganishia*, *Cutaneotrichosporon*, *Sporobolomyces*, *Sporodiobolus*, *Trichosporon*, and *Vanjira* spp.

### 2.3. Molds in Dairy Products

Dairy product spoilage by molds also consists in visible alterations due to the outgrowth of a thallus on the product surface. In vacuum-packed cheeses such as cheddar cheese, another defect called thread mold defect is sometimes encountered and is characterized by mold growth in folds and wrinkles of the plastic film in which the cheese is packaged [54]. Mold spoilage can also lead to the formation of off-flavors. For example, *Moniliella suaveolens* and *Cladosporium herbarum* were shown to produce ketones causing rancidity defect in butter while *Penicillium glabrum*, *Penicillium expansum*, *Penicillium chrysogenum*, and *Cladosporium cladosporioides* produced off-flavors including 2-methylisoborneol and geosmin which possess strong musty and earthy notes [2]. Interestingly, a study [58] showed that certain fungal species were able to metabolize sorbate salts (used as preservatives) to produce metabolites such as *trans*-1,3-pentadiene or *trans*-piperylene, resulting in the formation of plastic-like or “kerosene” off-flavors. 

Molds responsible for dairy product spoilage are highly diverse at the genus and species levels (Table 2). Indeed, up to 100 mold species have been identified so far as being responsible for dairy product spoilage. Most species belong to the Ascomycota and Mucoromycota phyla (Table 2). Both Ascomycota and Mucoromycota have been principally isolated from hard or semi-hard cheeses. 

Among Ascomycota, all identified spoilage molds (88 species distributed in 26 genera) belong to the Pezizomycotina subdivision, with the exception of *Lecanicillium lecanii* [59]. Within the Pezizomycotina subdivision, the Eurotiomycetes class is the most represented (nine genera and 57 species), followed by the Dothideomycetes (10 genera and 20 species), the Sordariomycetes (11 species of six genera) and the Leotiomycetes (one species). Among Eurotiomycetes, *Penicillium* is by far the most frequently reported spoilage genus with ~40 species, followed by *Aspergillus* (10 species). Other genera include *Byssochlamys*, *Eupenicillium*, *Eurotium*, *Exophiala*, *Hamigera*, *Neosartorya*, *Rhinocladiella*, and *Talaromyces* spp. 

*Penicillium* spp. are mainly isolated from hard and semi-hard cheeses but are also found in all other product types including fresh, blue-veined, mold-ripened, soft- and semi-soft cheeses, butter, yoghurt, milk as well as in buffalo, goat, or ewe’s milk cheeses. Based on the classification system recently proposed by Houbraken and Samson [60], *Penicillium* spp. responsible for spoilage mainly belong to the Fasciculata section (11 species) but also to the Chrysogena (three species), Biverticillium (four species), Brevicompacta (three species) and Aspergilloides (two species) sections. Among the Fasciculata section, *Penicillium commune*, *Penicillium solitum*, *Penicillium crustosum*, and *Penicillium verrucosum* are frequently reported, while within the Chrysogena section, *P. chrysogenum*, *Penicillium nalgiovense*, and *Penicillium griseofulvum* are quite common. Within Brevicompacta, *Penicillium brevicompactum* is frequently reported, in addition to *Penicillium bialowiezense* [49] and *Penicillium spathulatum* [61]. Nonetheless, Samson et al. [62] underlined that *P. bialowiezense* could have been misidentified in previous studies with the closely related species *P. brevicompactum*. The *Penicillium* species mentioned above are adaptive to low temperatures, and several of them are considered xerophilic species [2], therefore, their occurrence in spoiled dairy products is not surprising. *Penicillium* spp. is also well-adapted to the cheese environment and has been associated with cheese manufacturing for centuries. In addition, a recent study highlighted the presence of not previously mentioned xerophilic species such as *Penicillium charlesii*, *Penicillium fellutanum*, *Penicillium adametzioides*, and *Penicillium antarcticum* [49]. 

Fungi from the Dothideomycetes class are principally isolated from hard and semi-hard cheeses and constitute the second most frequently identified class after Eurotiomycetes. Among this class, *Cladosporium*, with seven species distributed in three different *Cladosporium* complexes, is the most frequently reported. *Cladosporium* spp. grow slowly but are very common airborne fungi and are quite psychrotolerant and xerotolerant [2]. 

Among the Mucoromycotina sub-phylum, three different genera have already been isolated from contaminated dairy products, i.e., *Mucor* (four species), *Rhizopus* (one species), and *Thamnidium* (one species), all belonging to the Mucorales order. As for other genera belonging to the Ascomycota phylum, these genera were mostly isolated from spoiled cheeses [56,62,63]. 

Molds can also contaminate heat-treated dairy products. Indeed, species belonging to *Aspergillus*, *Byssochlamys*, *Cladosporium*, *Eupenicillium*, *Fusarium*, *Hamigera*, *Neosartorya*, *Penicillium*, and *Talaromyces* genera have been isolated from pasteurized milk, cream cheese, and heat-treated dairy beverages [2,49,57]. The presence of mold in heat-treated milk or dairy products might be due to post-processing contaminations during bottling or packaging [57] or to the heat-resistance of mold spores. Indeed, Pitt and Hocking (2009) isolated heat-resistant species such as *Byssochlamys nivea* (anamorph *Paecilomyces niveus*), *Eupenicillium brefeldianum*, *Hamigera avellanea*, and *Neosartorya fischeri* (anamorph *Aspergillus fischeri*) from cream cheese, as well as *B. nivea*, *N. fischeri*, and *Talaromyces macrosporus* from heat-treated dairy beverage [2]. In a recent study, it was shown that the dormancy of *Talaromyces* and *Neosartorya* spp. ascospores was broken during thermal processing, for which heat-treatment was not optimized for ascospore inactivation [64].

In addition, fungal spoilage of dairy products obtained by Ultra-High Temperature (UHT) treatment can also occur, but it is generally due to post-processing contaminations. For example, *Fusarium oxysporum* was isolated on several occasions from UHT-flavored milk beverages [2]. 

## 3. Prevention and Control of Fungal Spoilage in Dairy Products

**Figure 1:**
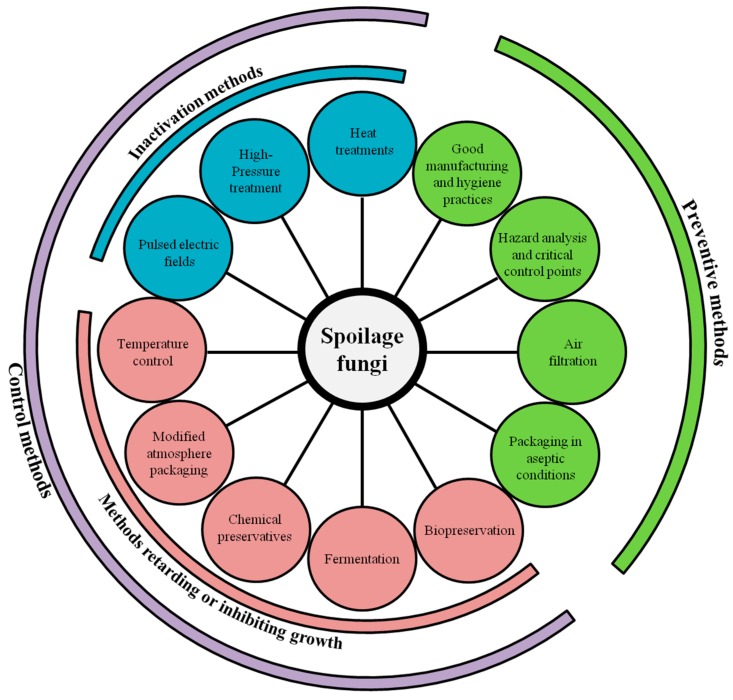
Preventive and control methods used in dairy products for preventing, inactivating, retarding, or inhibiting growth of spoilage fungi.

Today, fungal spoilage continues to be a major challenge for industrials and, in modern practices, both preventive and control approaches are usually combined to reduce its incidence in dairy foods (Figure 1). Preventive methods can be defined as methods which can avoid contamination or recontamination during product processing, including packaging in aseptic conditions, use of air filtration system, and good manufacturing practices such as good hygienic practices and Hazard Analysis and Critical Control Points (HACCP) system implementation [71]. Control methods involve the use of methods which will either slow down or inhibit microbial growth, such as the addition of chemical preservatives, use of modified atmosphere packaging, and low temperature storage, as well as methods which will inactivate fungi such as heat and high-pressure treatments [71]. 

When it comes to choosing the most appropriate preventive and control methods, different factors must be taken into account. One major factor is the characteristics of the product to be preserved (composition, a_w_) and those of the microorganisms of concern as well as the sanitation conditions along the manufacturing process and product storage conditions. Another important factor is also consumer perception and acceptance of food preservation methods as well as their impact on hygiene and safety and their associated cost [72,73]. That is probably the reason why, among emerging methods to combat fungal spoilage, the use of bioprotective cultures and fermentates to decrease the use of chemical preservatives is gaining more and more attention [74].

### 3.1. Preventive Methods

#### 3.1.1. Good Manufacturing and Distribution Practices

The success of any food preservation method depends on low initial levels of contamination, which in turn are achieved through the application of appropriate cleaning, sanitation, decontamination, and hygienic practices [73]. Dairy product manufacturing and packaging must also be done in the most hygienic and aseptic conditions possible to reduce the risk of biological contamination including that of fungal contamination [75,76]. In a code of good manufacturing and distribution practices (GMDPs), two main points are essential: (1) selecting good quality raw materials and monitoring the manufacturing process to control microbes while preventing cross-contamination; and (2) avoiding or retarding microbial growth [77,78]. GMDPs are an indispensable part of every food quality system. This code relies on the use of good hygiene practices and on the implemented hazard analysis critical control point (HACCP) systems from the production to the consumption of dairy products. In accordance with the *Codex Alimentarius* indications and Regulation 852/2004/CE, HACCP systems identify, evaluate, and control hazards that threaten food hygiene, in particular taking into account the microbiological risks [79,80]. Thus, the microbiological quality of dairy products is controlled at each step of the production process, from the raw materials to the finished product [81]. Prerequisite programs, such as Total Quality Management (TQM), a method including planning, organizing, and understanding each activity and involving each individual at each level, or Just-In-Time (JIT), a program designed to reduce waste by reducing flow times within the production system as well as response times from suppliers and to customers, are useful to improve product quality [81,82]. These programs have significant costs and require important efforts, but will produce return in investment in the long term. 

#### 3.1.2. Air Filtration and Decontamination Systems

With specific regard to the fungal contamination risk, potential contamination sources include ambient air. Therefore, efficient air-filtration systems should be in place to reduce spore counts into the air where the product is vulnerable. For example, Beletstiotis et al. [29] showed that the operation of High Efficiency Particulate Air (HEPA) filters class 10.000, designed to withhold fungal spores, allowed a decrease in fungal loads by 30 times in the indoor air of a dairy plant. The direction of air flows and location of outlets should also be carefully examined in sensitive zones. Moreover, the control of air pressurization can be applied to prevent air flow from the dirtiest to cleanest areas. Cleanroom technologies (aseptic or ultraclean technologies) including minienvironments that protect equipment such as filling and packaging equipment, transport system tunnels, or protective enclosures can also be used. 

For air decontamination, gaseous ozone was successfully applied for disinfecting empty cheese ripening and storage facilities with up to a 10-fold reduction in mold and yeast counts depending on the dose and treatment time [83,84], see for a review Varga and Szigeti, 2016 [85]. Finally, molds and yeasts are able to grow in humid zones (walls, ceiling, and floor) and on the equipment if these are not properly cleaned and disinfected. 

Various disinfectants such as alcohol, peracetic acid, iodophors, aldehydes, quaternary amine compounds, chlorine-based agents, or hydrogen peroxide have been used in the dairy industry against fungal contaminations. Nonetheless, fungal response to such cleaning agents varies with disinfectant type and concentration. For example, Korukluoglu et al. [86] showed that *Aspergillus niger* was sensitive to alcohol but not to peracetic acid; moreover, *A. niger* was resistant to 0.5% iodophor but sensitive at 1%. In addition, Bundgaard-Nielsen and Nielsen (1995) demonstrated the existence of intraspecific variation in tolerance to quaternary ammonium compounds, especially in *P. commune* and *Penicillium nalgiovense* [87]. Finally, Martin et al. [88] revealed that the combination of hydrogen peroxide and formic acid had a synergistic action against *Scopulariopsis brevicaulis* and *G. geotrichum*, thus showing that a combination of different disinfectants is a possible solution to prevent fungal contaminations. More work is required to investigate the efficiency of disinfectant agents against fungi in the dairy product context, while taking into account the continuously evolving legislation on their use.

### 3.2. Control Methods

#### 3.2.1. Inactivation Treatments

Concerning heat-treatments, thermization, pasteurization, and UHT sterilization are intended to partially or completely reduce milk microbial load while altering as little as possible milk organoleptic and nutritional properties [89]. During such heat-treatment, the product is subjected to a high temperature for a short period of time. In the EU, thermization, pasteurization, and UHT correspond to treatments at 57–68 °C for a minimum of 15 s, 71.7 °C for 15 s, and at least 135 °C for a few seconds, respectively [90]. According to Sakkas et al. [91], the efficacy of heat treatments are related to the temperature–time combinations, heating method utilized, and milk pre-treatment conditions (filtration, homogenization, standardization, etc.). Concerning milk thermization, the main objective is to reduce the population of spoilage microorganisms and to inactivate enzymes while minimizing heat damage to milk components. Milk pasteurization is much more efficient than thermization, but not all microorganisms can be destroyed and this treatment has to be followed by a rapid chilling (4 °C) to slow down subsequent microbial growth. Finally, UHT sterilization, used for milk and dairy drinks, is itself obtained by applying a continuous heat-flow for a short time-period which allows for the total destruction of microorganisms followed by milk aseptic packaging [87]. 

As mentioned earlier, certain fungal species, because of their considerable heat-resistance, are able to spoil heat-treated dairy products, in particular pasteurized fruit-based products. Heat-resistant species include *Byssochlamys spectabilis*, *N. fischeri*, *H. avellanea*, and *Talaromyces bacillisporus* [92,93]. This feature is due to their heat-resistant ascospores which are activated when heat-treatments are not high enough to inactivate them. As an example, *N. fischeri* and *T. bacillisporus* were shown to possess decimal reduction times at 85 °C (D_85_) ranging from 15.0 to 30.1 min, and from 11.9 and 15.5 min, respectively [39,94]. *N. fischeri* and *T. bacillisporus* showed D_85_ ranging from 47 to 75 min in *N*-(2-Acetamido)-2-aminoethanesulfonic acid buffer as well as D_90_ ranging from 7.5 to 12.7 min and D_95_ ranging from 0.56 to 0.67 min in blueberry juice [95]. They showed D_90_ from 2.0 to 7.6 min and D_95_ from 1.7 to 2.3 min in juices. *N. fischeri* and *T. bacillisporus* showed D_87_ from 11.1 to 66.7 min, D_90_ from 4.7 to 13.5 min, and D_95_ from 0.43 to 1.52 min and ranged between 44.4 and 60.9 min at 82 °C and 2.7 and 4.1 min at 88 °C. As another example, *H. avellanea* ascospores are activated but not destroyed after heat-treatment for 20 min at 87 °C, 5 min at 90 °C, and 1 min at 95 °C, showing the importance of time-temperature conditions treatment [93]. 

Other techniques such as high-pressure (HP) treatments of dairy products can also be used to inactivate yeast cells and mold spores (among other microorganisms) and extend product shelf-life [95]. Mainville et al. [96] and Reps et al. [97] showed that yeasts from kefir were completely inactivated at 400 MPa for 15 min or 30 min without significant changes in protein and lipid structure of the product. Moreover, Evert-Arriagada et al. [98] recently demonstrated that HP processing under industrial conditions could extend the shelf-life of a commercial starter-free fresh cheese. Pressurized cheeses were firmer and more yellow than control cheeses, but these changes did not affect the panel preference for pressurized cheese. It is worth mentioning that there is an increasing number of HP-treated dairy products available on the market as this technology meets consumer’s expectations for more “natural” and less-heavily processed food products.

Pulsed electric fields (PEF) treatment consists of pulsed electric fields of strong intensity (15 to 50 kV/cm) for only few seconds [99]; this represents another emerging technology for microbial inactivation, including that of yeast and molds, with potential application in fluid dairy foods (see the recent reviews of Buckow et al. [100] and Wan et al. [101]). 

#### 3.2.2. Temperature Control

Temperature control is critical for dairy food quality and shelf-life. Cold temperatures are used to minimize microbial growth in raw milk until processing and to extend the shelf-life of non-sterile dairy foods. Low temperatures, or even frozen storage, however, do not eliminate microorganisms [73]. Milk and dairy products are generally stored between 0 °C and 4 °C and at temperatures ranging from 4 to 10 °C at the consumer’s home. However, most fungi are psychrotrophic and are thus able to grow at such temperatures. In a recent study, Gougouli et al. [102] evaluated the effect of temperature and inoculum size on the growth of one isolate each from 12 fungal species during yoghurt storage. The ability to grow at refrigerated temperature was species-dependent with estimated minimal growth temperatures of −6.7 and 9.6 °C for *P. commune* and *A. niger*, respectively. In addition, Gougouli et al. [102] also developed and validated a probabilistic model to predict the appearance of visible mycelia originating from single spores which could be used for choosing adequate conditions for challenge tests. It should be noted that besides storage temperature and the spoilage organism itself, fungal spoilage susceptibility depends on the spoiler initial population and the time required to form a visible thallus on the product surface, which can be predicted using predictive mycology tools. 

#### 3.2.3. Modified Atmosphere Packaging

Modified atmosphere packaging (MAP) is a commonly used method to control fungal spoilage and thus to extend dairy food shelf-life. MAP consists of the replacement of air by a defined gas mixture. In MAP, active agents such as carbon dioxide [103] or oxygen scavengers [104] can also be utilized. During storage, passive modifications of the headspace gases can occur, resulting from product respiration and gas transfer through the film. 

As extensively reviewed by Nguyen Van Long et al. [105] on a large number of fungal species, lowering the oxygen level below 1% will result in the partial growth inhibition of strict aerobic fungi, while it will slow down facultative anaerobes’ growth as it enables O_2_ use as a final electron acceptor. However, for many species including *Penicillium* and *Mucor* spp., only 0% O_2_ concentration can totally inhibit growth [105]. It is worth mentioning that such an O_2_ level will favor the growth of facultative or strict anaerobes and that it can have a detrimental effect on products such as mold-ripened cheeses, for which a certain oxygen level is necessary to maintain their organoleptic properties [106]. An increase of the CO_2_ level in package atmosphere can have an inhibitory effect, thus further extending product shelf-life [105]. In their extensive review, Nguyen Van Long et al. [105] indicated that, independent of the fungal species, a CO_2_ level above 50% was necessary to achieve fungal growth inhibition and that, depending on the fungal species, 50–90% were necessary to completely inhibit fungal growth in solid matrices. Three mechanisms have been described to explain the CO_2_ antimicrobial effect. The first mechanism is the displacement of some or all of the O_2_ available for growth of aerobic microorganisms. The second one is a pH decrease resulting from CO_2_ dissolution into the product and carbonic acid formation. Carbonic acid will then behave as a weak organic acid provoking disturbances in pH homeostasis [107,108]. Nonetheless, high CO_2_ concentration may sometimes affect product organoleptic al properties. For example, high CO_2_ levels (100%) led to very negative effects in the sensory qualities of cottage cheese, whereas for others, such as cheddar, packaging with 100% CO_2_ is commonly used in dairy industries [109,110]. 

MAPs are often used for cheeses and different gas compositions have been suggested [109,111,112]. For example, in commercial sliced cheddar cheese, *P. commune* did not grow in atmospheres composed either of 20%, 40%, and 60% CO_2_ and less than 0.5% O_2_ [113]. *Eurotium chevalieri* and *Xeromyces bisporus* could not grow under atmosphere containing 80% CO_2_ and 20% O_2_ during incubation for 60 days, whereas *Mucor plumbeus* development was observed after 15 days [111]. Facultative anaerobic fungi are also susceptible to high CO_2_ levels [113,114,115]. As an example, a 60% CO_2_/40% N_2_ modified atmosphere was effective for inhibiting yeast growth and extending the shelf-life of whey cheese [113]. 

A good temperature control is necessary for effective MAP use because the CO_2_ effect is enhanced as the temperature decreases. A combination of refrigeration and the storage of milk and milk products under modified gas atmospheres for the extension of shelf life has been reviewed extensively [108,116,117,118]. In general, most of the used atmospheres do not completely inhibit growth, but the spoilage time is delayed. Overall, more research is needed to improve our knowledge on O_2_ and CO_2_ effects on the germination and growth of spoilage fungi and, thus, to improve MAP efficiency. 

#### 3.2.4. Chemical Preservatives

In addition to the methods mentioned above, chemical preservatives are widely used in the dairy industry. Preservatives are food additives that help to prevent dairy products from spoilage by bacteria and/or fungi. Antifungal preservatives used in dairy products include weak organic acids, such as sorbic acid, benzoic acid, and propionic acid, and their salts, such as potassium sorbate, calcium sorbate, sodium benzoate, potassium benzoate, calcium benzoate, and sodium propionate, as well as natamycin, a polyethylene antibiotic (Table 3).

Weak acids inhibit both bacterial and fungal growth. In addition, sorbic acid also inhibits spore germination in bacteria [121,122]. Preservatives such as benzoic and sorbic acid have an optimal inhibitory activity at pH between 4.5 and 5.5. Indeed, at such pH, the uncharged, undissociated acid form can diffuse freely across the cytoplasmic membrane and enter into the cell. The equilibrium between the undissociated and dissociated forms depends on the acid pKa and the medium pH. Once inside the cell (pHi ~ 7), the acid will dissociate and release charged anions and protons which cannot diffuse back across the membrane. Anions and protons accumulation into the cell is responsible for intracellular pH decrease and may cause membrane disruption, inhibition of essential metabolic reactions, stress in intracellular pH homeostasis, and/or accumulation of toxic anions which finally lead to cell death [14]. In a recent study, Garnier et al. [49] determined the minimal inhibitor concentration (MIC) of different weak acids including potassium sorbate, calcium propionate, and sodium benzoate for several fungal strains isolated from spoiled dairy products. They found that, independent of the studied species, calcium propionate was the least effective preservative followed by sodium benzoate and potassium sorbate. It should be emphasized that the inhibitory effect of weak acids on fungi is also influenced by other factors such as a_w_. For example, the MIC of sorbic acid were 0.1% and 0.15% (*w*/*w*) at 0.85 a_w_ while they were 0.075% and 0.05% (*w*/*w*) at 0.90 a_w_, for *Aspergillus flavus* and *P. roqueforti*, respectively [123]. Moreover, Stratford et al. [124] recently showed that, in contrast to acetic acid, sorbic acid did not decrease pHi in *S. cerevisiae* but instead was a membrane-active compound, inhibiting the activity of a plasma-membrane H^+^-ATPase proton pump. Most studies on the action mechanism of weak acids have been performed on *S. cerevisiae* and *Zygosaccharomyces bailli*. Therefore, more studies are necessary to understand the exact inhibition mechanism of weak acids against spoilage fungi encountered in dairy products. Finally, for hard and semi-hard smear cheeses, which are characterized by the development of a red-orange microbial mat on their surfaces, films permeable to moisture and O_2_ can be utilized to pack the cheeses in order to protect them from yeast and molds present in the dairy environment. Such films can also be coated with antifungal molecules such as natamycin. It is worth mentioning that a successful attempt has been made to replace synthetic film polymers by a chitosan coating containing natamycin for protecting semi-hard cheese from fungal contamination [125].

Fungal resistance or adaptation to weak organic acids rely on several mechanisms. In *S. cerevisiae* and other fungi, it includes maintenance of the cell wall structure, changes in plasma membrane or cell wall composition, metal metabolism, and activation of ATP-consuming membrane transporters to remove protons and anions [126]. Ullah et al. [127] showed that *S. cerevisiae* adaptation to sorbic or acetic acid resulted in a decreased diffusional entry of the molecule. They concluded that pre-exposed cells indeed decreased acid entry through alteration of either the plasma membrane structure or the cell wall composition or through an increase in intracellular buffering capacity. In addition, Brandao et al. [38] showed that the H^+^ internal concentration in *S. cerevisiae* was regulated by several systems, including plasma membrane H^+^-ATPase, and that ENA1p, known for its in involvement in saline or alkaline stress responses and regulation of the plasma membrane potential had an important but yet to be fully understood role in the cellular response to acid [126]. This study also demonstrated that acid stress response was dependent on calcium metabolism and was blocked by a calcineurin inhibitor. Other resistance mechanisms include the metabolization of weak acids into the cell. For example, Casas et al. [128] showed that *D. hansenii* could metabolize potassium sorbate into pentadiene, a volatile compound also responsible for off-odor, while *A. niger* was able to decarboxylate and detoxify sorbic acid thanks to a phenylacrylic acid decarboxylase [129].

Natamycin (also known as pimaricin) is a fungicide belonging to polyethylene antibiotics [130]. It is currently used to control fungal growth on cheese surfaces [131,132,133]. Produced by *Streptomyces natalensis*, this antibiotic acts by inhibiting the vacuole fusion process through specific interaction with ergosterol at the early priming stage of fusion, but does not permeabilize the membrane. 

A World Health Organization (WHO) monograph on Food Additives states that obtaining resistance against natamycin is difficult, because of the action mode of these chemical agents [134]. Indeed, natamycin binds to ergosterol, an important component of the plasma membrane and of the growing tips of germinating spores and vegetative hyphae [135,136]. In addition, natamycin MIC of different fungal species were reported to only differ negligibly, which is not in favor of resistance development [134]. For example, *C. parapsilosis*, *Rhodotorula* spp., and *Penicillium* spp. had mean MIC of 5.2 µg/mL, 2.3 µg/mL, and 2.3 µg/mL, respectively [137]. In contrast, Garnier et al. [49], who evaluated natamycin MIC after surface-treatment of one isolate each of 10 species from spoiled dairy products, observed that MIC ranged from 0.04 mg/dm^2^ in *Cladosporium halotolerans* to >0.2 mg/dm^2^ in *Y. lipolytica*. In addition, a recent study [138] showed that a continuous and prolonged incubation with natamycin induced a tolerance in individual strains. 

The WHO [139], European Food Safety Authority [119], and Food and Drug Administration [120] have evaluated natamycin thoroughly and all list it as safe for human consumption. In the US, according to the Code of Federal Regulations, the final amount of natamycin must not exceed 20 ppm in cheese (20 mg/kg). In the EU, natamycin is only allowed for cheese surface-treatment with a maximum applied dose of 1 mg/dm^2^. Moreover, it must not be present at a depth >5 mm in the finished product [140].

#### 3.2.5. Fermentation

Fermentation is one of the oldest preservation methods [141,142]. It is a biological process relying on the activity of specific microorganisms producing certain metabolites with antifungal and antibacterial activities [12,141]. Different microbial groups involved in dairy product manufacturing may suppress or retard the growth of spoilage fungi. LAB produce lactic acid as a major fermentation end-product, but may also produce other metabolites with antifungal activity (see also Section 4). Certain cheese types such as smear cheeses and mold-ripened cheeses also harbor on their surfaces complex microbiota composed of desirable aerobic bacteria and fungi which can compete with spoilage fungi for one or more limiting macro- and/or micronutrients and/or for space. 

A complementary approach to reduce fungal spoilage in dairy products is gaining more and more interest: the use of bioprotective cultures and fermentates.

## 4. Bioprotective Cultures

Today, in more economically developed countries, there is a strong and increasing demand from consumers for foods that are more “natural”, i.e., less heavily processed and preservative-free [75,143]. That is the reason why a strong market demand exists for natural solutions to ensure both food safety and food shelf-life [144]. Biopreservation is not a new concept as it has been used for thousands of years in fermented foods. Also called biocontrol, it refers to the extension of food shelf-life and increase in food safety using natural or added microbiota and/or their antimicrobial compounds [145]. Food bioprotective cultures can thus be defined as food-grade bacterial or fungal strains that have been selected for their antimicrobial properties. They differ from starter or adjunct cultures which are primarily used for their technological functions (acid and aroma production, role in texture, color, etc.). Among microorganisms possessing antimicrobial properties, LAB, produce a large array of antimicrobial substances including organic acids such as lactic and acetic acids, fatty acids, reuterin, antifungal peptides, and bacteriocins [146,147]. While research has mainly focused on screening for bacterial strains with antimicrobial activity against pathogenic or spoilage bacteria and fungi, fungi with antimicrobial activities also exist [148]. 

### 4.1. Lactic Acid Bacteria and Propionibacteria with Antifungal Activity

LAB and propionibacteria (PAB) can be used in food production as starter cultures to modify and improve nutritional and organoleptic food properties, or as protective cultures to improve product safety and/or shelf-life [144,149]. Indeed, LAB are used as starter cultures in the manufacturing of dairy products such as fermented milk, yoghurt, buttermilk, cottage cheeses, hard cheeses, and soft cheeses, among many others [150]. LAB have also been traditionally used as natural biopreservatives of food and feed, including milk and dairy products. 

Antifungal LAB have been studied in a large food range including dairy products [15,151]. Today, several recent studies report the use of antifungal LAB to control dairy product spoilage [15,152,153]. LAB produce many antifungal metabolites [154] and most LAB are granted with a generally recognized as safe (GRAS) and qualified presumption of safety (QPS) status. Besides being safe for human consumption (absence of biogenic amine production and acquired antibiotic resistance), the main properties expected from antifungal LAB and PAB bioprotective cultures are: (1) an antifungal activity exhibited and maintained during manufacturing and storage; (2) no impact on starter cultures’ functionalities; (3) no modification of product organoleptic properties; (4) an activity at the lowest possible inoculum to reduce the cost associated with their use; and (5) an easy propagation at high populations and resistance to lyophilization or freezing. There are currently several antifungal bioprotective cultures commercially available for dairy products, such as HoldBac series (DuPont Danisco), FreshQ^®^ series (Dupont), and Befresh^TM^ AF (Handary). Holdbac YM-B is a mixed culture of *Lactobacillus rhamnosus* and *Propionibacterium freudenreichii* subsp. *shermanii* and FreshQ^®^ 2 is a single *L. rhamnosus* culture, while Befresh^TM^ AF is a mixture of *Lactobacillus paracasei* and *P. freudenreichii* subsp. *shermanii*. Despite such bioprotective cultures being available on the market, research for new LAB with antifungal activities and identification of their associated metabolites is now the focus of many academic and industrial research groups.

Screening for antifungal strains is a critical step among those required to develop antifungal cultures. For example, a recent study described a high-throughput screening method to detect antifungal activities in *Lactobacillus* species cultivated in Man, Rogosa, and Sharpe (MRS) media [155]. This method allowed the detection of 154 strains with antifungal activities against *R. mucilaginosa* and to a lower extent against *Aspergillus tamari*, *Candida krusei*, and *K. marxianus*. However, as mentioned in several papers [156,157], screening in conventional MRS medium is not recommended as MRS contains acetate which may potentialize antifungal activity and artificially inflate the number of active isolates. Another interesting but labor-consuming approach, used by Delavenne et al. [158] to develop an antifungal isolate collection, was to plate raw milk samples on eight semi-selective media for LAB, and to systematically screen colonies for their antifungal activity against four spoilage fungi using the agar-overlay method. Among the ~72,000 tested colonies, >−1200 colonies (i.e., 1.7% of tested colonies) had a detectable antifungal activity. However, one should keep in mind that laboratory media differ significantly in their physicochemical and microbiological characteristics with those of dairy foods and, thus, active strains in laboratory media may lose this ability in real products. Overall, further work is needed to develop high-throughput screening methods in dairy products mimicking models to increase the chances of finding suitable strains. 

Among antifungal LAB, *Lactobacillus* and, to a smaller extent, *Lactococcus*, *Pediococcus*, *Weissella*, and *Leuconostoc* are the most frequently cited genera (Table 4). Indeed, many strains pertaining to species of the *Lactobacillus* genus including *Lactobacillus plantarum*, *Lactobacillus casei*, *Lactobacillus paracasei*, and *Lactobacillus brevis* were shown to possess antifungal activity against a large spectrum of fungal targets including *Penicillium*, *Candida*, *Kluyveromyces*, and *Rhodotorula* spp. as well as *Debaryomyces hansenii* and *Yarrowia lipolytica*, which are among the most important spoilage agents in dairy products [49]. Among *Propionibacterium*, *P. freudenreichii* is the major species reported to possess antifungal activity [157,159].

So far, besides lactic, acetic, and propionic acids, which are produced at g/L or g/kg levels, a very large variety of molecules has been reported to be responsible for antifungal activity. These molecules are generally produced at lower levels (mg/L or mg/kg), and include organic acids (2-pyrrolidone-5-carboxylic acid, 3-phynyllactic acid, 4-hydroxybenzoic acid, azelaic acid, dl-Þ-hydroxyphenyllactic acid, hydroxyphenyllactic, *p*-coumaric acid, phenyllactic acid (*S*)-(−)-2-hydrocinnamic acid, succinic acid, vanillic acid), fatty acids (3-hydroxydecanoic acid, decanoic acid, hydroxyisocapric acid), cyclopeptides (cyclo(l-Pro-l-Pro), cyclo(l-Leu-l-Pro), cyclo(l-Tyr-l-Pro) cyclo(l-Met-l-Pro), cyclo(Phe-Pro), cyclo(Phe-OH-Pro), cyclo(l-Phe-LPro), cyclo(l-Phe-*trans*-4-OH-l-Pro) and cyclo(l-His-l-Pro)), reuterin [160], and volatile compounds such as diacetyl [153] (Table 4). Because these molecules are produced at quantities below their MIC, it is likely that they act in synergy. For example, in a study focusing on the effect of organic acids on molds, Dagnas et al. [161] showed that lactic acid alone had almost no inhibitory effect against several mold species while lactic and acetic acids could act in synergy.

Similar results were reported for other organic acids such as PLA, in which MIC decreased in the presence of lactic acid [185]. Only few studies [154,197] dealt with the action mode of LAB antifungal metabolites and the response of fungi at the physiological, transcriptomic, or proteomic levels. Therefore, further research should be undertaken in this area. 

As shown in Table 3, most studies on LAB and PAB antifungal activity have been performed in (semi-)synthetic culture medium and it is obvious that important discrepancies can exist between in vitro and in situ tests. The main reasons for this are that intrinsic and extrinsic factors can affect the production of antifungal metabolites, their activity, and the susceptibility of the fungal target to these compounds. Intrinsic factors include medium composition in terms of macro- and micronutrients, pH, a_w_, Eh, and food structure, while extrinsic factors are temperature, composition of the surrounding atmosphere, and humidity. Therefore, a second critical step in developing antifungal cultures is to test their efficiency in real products using challenge- and durability-tests and against one or several fungal targets. It should be noted that only a few publications have clearly shown in situ antifungal activity of selected LAB and PAB (Table 4). For example, Delavenne et al. [15,27] showed the antifungal activity of *Lactobacillus harbinensis* KV931Np against six fungal targets in yoghurt while Schwenninger and Meile (2004) demonstrated the antifungal activity of three mixed cultures of *Lactobacillus paracasei* subsp. *paracasei* SM20, SM29, or SM63 and *Propionibacterium jensenii* SM11, against *Candida pulcherrima*, *C. magnoliae*, *C. parapsilosis*, and *Zygosaccharomyces bailii* in yoghurt and on cheese surfaces [151]. More recently, Aunsbjerg et al. [153] proved the antifungal activity of *Lactobacillus paracasei* in chemically defined medium and in yoghurt against *P. solitum* and *Penicillium* sp.

### 4.2. Fermentates

LAB and PAB may also be used to produce dairy fermentates containing antifungal metabolites, which are fermented dairy ingredients produced from milk via a fermentation process [144]. MicroGARD (DuPont Danisco) and DuraFresh (Kerry) are two currently available commercial fermentates. As an example, MicroGARD, which is FDA-approved, is produced by skimmed milk fermentation using *P. freudenreichii* subsp. *shermanii*. The use of this fermentate in cottage cheese partially inhibited *K. marxianus* and *P. expansum* and thus extended its shelf-life [197]. It may also be used in sour cream, yoghurt, and dairy desserts [198]. 

## 5. Conclusions

Despite technological advances, fungal spoilage is still a main issue in the dairy industry. Among the actual methods in use, a large focus concerns the replacement of traditional hurdle technologies such as chemical preservatives by new techniques to meet the increasing consumer demand for less-heavily processed and preservative-free dairy products. These new techniques include preventive methods such as a better management of air quality and non-thermal control methods such as modified atmosphere packaging and biopreservation using antifungal bioprotective cultures or fermentates. 

## Figures and Tables

**Table 1:** Diversity of spoilage yeasts in dairy products.

Phylum	Species	Product Types	References
Ascomycota	*Atelosaccharomyces pseudotropicalis*	Yoghurt	[26]
	*Blastodendrion arztii*	Fresh unripened cheese	[51]
	*Candida acidothermophilum*	Yoghurt	[26,36,52]
	*Candida apis*	Hard or semi-hard cheese	[53]
	*Candida beverwijkiae*	Hard or semi-hard cheese	[51]
		Yoghurt	[26]
	*Candida blankii*	Heat-treated milk and dairy product	[53]
	*Candida glabrata*	Soft and semi-soft cheese	[51]
	*Candida glaebosa*	Hard or semi-hard cheese	[54]
	*Candida inconspicua*	Buffalo, goat, or sheep cheese	[51]
		Fresh unripened cheese	[49,51]
		Soft and semi-soft cheese	[51]
	*Candida intermedia*	Hard or semi-hard cheese	[2,54]
		Other dairy products	[49]
		Yoghurt	[26]
	*Candida krusei*	Soft and semi-soft cheese	[26]
	*Candida lusitaniae*	Buffalo, goat, or sheep cheese	[51]
		Fresh unripened cheese	[51]
		Soft and semi-soft cheese	[51]
		Yoghurt	[26,52]
	*Candida multigemmis*	Hard or semi-hard cheese	[2,54]
	*Candida mycoderma*	Buffalo, goat, or sheep cheese	[51]
	*Candida norvegica*	Soft and semi-soft cheese	[11]
	*Candida parapsilosis*	Fresh unripened cheese	[51]
		Hard or semi-hard cheese	[2,51,54]
		Soft and semi-soft cheese	[11,49,51]
		Yoghurt	[2,49]
	*Candida pseudoglaebosa*	Raw milk	[27]
	*Candida rugosa*	Soft and semi-soft cheese	[11]
	*Candida saitoana*	Yoghurt	[26]
	*Candida sake*	Fresh unripened cheese	[55]
		Hard or semi-hard cheese	[54]
	*Candida sphaerica*	Fresh unripened cheese	[26]
	*Candida stellata*	Hard or semi-hard cheese	[53]
		Yoghurt	[26,53]
	*Candida versatilis*	Yoghurt	[26,36]
	*Candida zeylanoides*	Fresh unripened cheese	[51]
		Hard or semi-hard cheese	[51]
		Soft and semi-soft cheese	[51]
	*Candida* sp.	Mold ripened cheese	[26]
	*Cyberlindnera jadinii*	Soft and semi-soft cheese	[11]
	*Debaryomyces hansenii*	Blue-veined cheese	[26]
		Buffalo, goat, or sheep cheese	[51]
		Fresh unripened cheese	[51,53,55]
		Hard or semi-hard cheese	[26,49,51]
		Heat-treated milk and dairy product	[26,52,53]
		Other dairy products	[52,53]
		Soft and semi-soft cheese	[11,51]
		Mold ripened cheese	[26]
		Yoghurt	[2,26,36,52,53]
	*Dekkera anomala*	Fresh unripened cheese	[55]
		Yoghurt	[26]
	*Exophiala* sp.	Fresh unripened cheese	[49]
	*Galactomyces geotrichum*	Butter and margarine	[2]
		Fresh unripened cheese	[2,49,55]
		Heat-treated milk and dairy product	[2]
		Other dairy products	[49]
	*Geotrichum capitatum*	Soft and semi-soft cheese	[51]
	*Geotrichum klebahnii*	Hard or semi-hard cheese	[51]
	*Geotrichum* sp.	Buffalo, goat, or sheep cheese	[56]
		Raw milk	[57]
	*Kazachstania unispora*	Soft and semi-soft cheese	[11]
	*Kluyveromyces lactis*	Fresh unripened cheese	[49,55]
	*Kluyveromyces marxianus*	Blue-veined cheese	[26]
		Fresh unripened cheese	[26,53]
		Hard or semi-hard cheese	[26,53,54]
		Heat-treated milk and dairy product	[26,53]
		Mold ripened cheese	[26]
		Other dairy products	[52]
		Raw milk	[27]
		Soft and semi-soft cheese	[49]
		Yoghurt	[2,26,36,53]
	*Meyerozyma guilliermondii*	Buffalo, goat, or sheep cheese	[51]
		Fresh unripened cheese	[49]
		Soft and semi-soft cheese	[11,51]
		Yoghurt	[49]
	*Naumovia dairenensis*	Soft and semi-soft cheese	[11]
	*Peterozyma toletana*	Yoghurt	[36]
	*Pichia fermentans*	Soft and semi-soft cheese	[11,49]
	*Pichia membranifaciens*	Fresh unripened cheese	[26]
		Soft and semi-soft cheese	[11]
	*Pichia norvegensis*	Soft and semi-soft cheese	[11]
	*Priceomyces carsonii*	Hard or semi-hard cheese	[54]
	*Saccharomyces cerevisiae*	Blue-veined cheese	[26]
		Buffalo, goat, or sheep cheese	[51]
		Fresh unripened cheese	[51,53]
		Hard or semi-hard cheese	[53]
		Heat-treated milk and dairy product	[26]
		Other dairy products	[52]
		Soft and semi-soft cheese	[11,51]
		Yoghurt	[26,36,53]
	*Torulaspora delbrueckii*	Fresh unripened cheese	[51]
		Soft and semi-soft cheese	[11]
	*Torulopsis* sp.	Yoghurt	[26]
	*Wickerhomomyces anomalus*	Soft and semi-soft cheese	[11]
		Yoghurt	[2]
	*Yarrowia lipolytica*	Blue-veined cheese	[26]
		Buffalo, goat, or sheep cheese	[51]
		Butter and margarine	[53]
		Fresh unripened cheese	[26,55]
		Hard or semi-hard cheese	[2,49,51,54]
		Mold ripened cheese	[2,26]
		Soft and semi-soft cheese	[11,49]
		Yoghurt	[2]
Basidiomycota	*Cryptococcus humicola*	Butter and margarine	[2]
		Hard or semi-hard cheese	[2]
	*Cryptococcus laurentii*	Butter and margarine	[53]
		Fresh unripened cheese	[26,53]
	*Cryptococcus pseudolongus*	Fresh unripened cheese	[49]
	*Cryptococcus* sp.	Heat-treated milk and dairy products	[52]
		Soft and semi-soft cheese	[11]
	*Cystobasidium minuta*	Soft and semi-soft cheese	[11]
	*Cutaneotrichosporon curvatus*	Raw milk	[26]
	*Cutaneotrichosporon cutaneum*	Hard or semi-hard cheese	[51]
		Fresh unripened cheese	[51]
	*Naganishia albida*	Butter and margarine	[53]
		Fresh unripened cheese	[53]
		Other dairy products	[53]
		Yoghurt	[53]
	*Rhodotorula diffluens*	Butter and margarine	[53]
		Fresh unripened cheese	[53]
		Hard or semi-hard cheese	[26,53]
		Heat-treated milk and dairy products	[26,52,53]
		Yoghurt	[2,26,53]
	*Rhodotorula glutinis*	Butter and margarine	[53]
		Fresh unripened cheese	[53]
		Other dairy products	[52,53]
		Soft and semi-soft cheese	[11]
	*Rhodotorula mucilaginosa*	Butter and margarine	[53]
		Fresh unripened cheese	[51,53]
		Other dairy products	[52,53]
		Soft and semi-soft cheese	[49]
		Yoghurt	[2,36]
	*Rhodotorula* sp.	Heat-treated milk and dairy products	[26]
	*Saitozyma flava*	Fresh unripened cheese	[53]
		Hard or semi-hard cheese	[53]
		Heat-treated milk and dairy products	[26,53]
		Other dairy products	[53]
	*Sporobolomyces roseus*	Fresh unripened cheese	[26]
	*Sporodiobolus salmonicolor*	Hard or semi-hard cheese	[49]
	*Trichosporon asahii*	Soft and semi-soft cheese	[49]
	*Vanrija humicola*	Fresh unripened cheese	[51]

**Table 2:** Diversity of spoilage filamentous fungi isolated from contaminated dairy products.

Phylum	Genera	Species	Product Types	References
Ascomycota	*Acremonium*	Nd *	Buffalo, goat, or sheep cheese	[56]
	*Alternaria*	*Alternaria alternata*	Blue-veined cheese	[63]
			Hard or semi-hard cheese	[54,65]
		Nd *	Hard or semi-hard cheese	[66]
			Raw milk	[25]
	*Aspergillus* section *Aspergillus*	*Aspergillus glaucus*	Heat-treated milk	[57]
			Raw milk	[57]
	*Aspergillus* section *Candidi*	*Aspergillus candidus*	Buffalo, goat, or sheep cheese	[56]
	*Aspergillus* section *Circumdati*	*Aspergillus ochraceus*	Buffalo, goat, or sheep cheese	[56]
	*Aspergillus* section *Flavi*	*Aspergillus flavus*	Hard or semi-hard cheese	[67]
			Heat-treated milk	[57]
			Raw milk	[57]
		*Aspergillus tamarii*	Blue-veined cheese	[63]
	*Aspergillus* section *Fumigati*	*Aspergillus fumigatus*	Blue-veined cheese	[63]
			Hard or semi-hard cheese	[65]
			Heat-treated milk	[57]
			Raw milk	[57]
	*Aspergillus* section *Nidulantes*	*Aspergillus sydowii*	Yoghurt	[2]
		*Aspergillus versicolor*	Blue-veined cheese	[63]
			Hard or semi-hard cheese	[59,68]
			Semi-soft cheese	[4,59]
			Buffalo, goat, or sheep cheese	[56]
	*Aspergillus* section *Nigri*	*Aspergillus niger*	Blue-veined cheese	[63,67]
	*Aspergillus*	Nd *	Hard or semi-hard cheese	[54,66]
			Raw milk	[25]
	*Aureobasisium*	*Aureobasidium pullulans*	Hard or semi-hard cheese	[69]
	*Bipolaris*	*Bipolaris australiensis*	Blue-veined cheese	[63]
	*Botrytis*	*Botrytis cinerea*	Hard or semi-hard cheese	[54]
	*Byssochlamys*	*Byssochlamys nivea*	Heat-treated milk	[2]
	*Cladosporium cladosporioides* complex	*Cladosporium*	Blue-veined cheese	[63]
		*cladosporioides*	Buffalo, goat, or sheep cheese	[56]
			Butter and margarine	[2]
			Hard or semi-hard cheese	[2,54,65]
			Buffalo, goat, or sheep cheese	[56]
		*Cladosporium phyllophilum*	Butter and margarine	[49]
	*Cladosporium herbarum* complex	*Cladosporium butyri*	Butter and margarine	[2]
		*Cladosporium herbarum*	Hard or semi-hard cheese	[2,54]
		*Cladosporium macrocarpum*	Hard or semi-hard cheese	[54]
	*Cladosporium sphaerospermum* complex	*Cladosporium halotolerans*	Heat-treated milk	[49]
		*Cladosporium sphaerospermum*	Hard or semi-hard cheese	[49,54]
	*Cladosporium*	Nd *	Hard or semi-hard cheese	[59,66]
			Semi-soft cheese	[59]
	*Didymella*	*Didymella pinodella*	Fresh unripened cheese	[49]
			Other dairy product	[49]
	*Epicoccum*	*Epicoccum nigrum*	Hard or semi-hard cheese	[54,69]
	*Eupenicillium*	*Eupenicillium brefeldianum*	Heat-treated milk	[2]
	*Eurotium*	*Eurotium herbariorum*	Hard or semi-hard cheese	[65]
		Nd *	Raw milk	[25,68]
	*Exophiala*	Nd *	Cream cheese	[49]
	*Fusarium*	*Fusarium avenaceum*	Buffalo, goat, or sheep cheese	[56]
		*Fusarium oxysporum*	Hard or semi-hard cheese	[54,59]
			Semi-soft cheese	[59]
			Heat-treated milk	[2]
		*Fusarium solani*	Hard or semi-hard cheese	[59]
			Semi-soft cheese	[59]
		*Fusarium verticillioides*	Hard or semi-hard cheese	[67]
		Nd *	Hard or semi-hard cheese	[66]
			Heat-treated milk	[57]
			Raw milk	[57]
	*Gliocladium*	*Gliocladium roseum*	Hard or semi-hard cheese	[54]
	*Hamigera*	*Hamigera avellanea*	Heat-treated milk	[2]
	*Lecanicillium*	*Lecanicillium lecanii*	Hard or semi-hard cheese	[59]
			Semi-soft cheese	[59]
	*Naumovia*	*Naumovia dairenensis*	Soft cheese	[11]
	*Neosartorya*	*Neosartorya fischeri*	Heat-treated milk	[2]
	*Penicillium* section	*Penicillium glabrum*	Butter and margarine	[2]
	*Aspergilloides*		Hard or semi-hard cheese	[2,54,70]
			Fresh unripened cheese	[2]
			Yoghurt	[2]
		*Penicillium spinulosum*	Hard or semi-hard cheese	[65]
	*Penicillium* section	*Penicillium funiculosum*	Buffalo, goat, or sheep cheese	[56]
	*Biverticillium*	*Penicillium minioluteum*	Hard or semi-hard cheese	[69]
		*Penicillium purpureogenum*	Hard or semi-hard cheese	[68]
		*Penicillium rugulosum*	Hard or semi-hard cheese	[54,59]
			Semi-soft cheese	[59]
	*Penicillium* section	*Penicillium bialowiezense*	Hard or semi-hard cheese	[49]
	*Brevicompacta*		Yoghurt	[49]
		*Penicillium brevicompactum*	Hard or semi-hard cheese	[2,49,54,59,65,69,70]
			Semi-soft cheese	[59]
			Buffalo, goat, or sheep cheese	[56]
	*Penicillium* section *Sclerotiora*	*Penicillium adametzioides*	Fresh unripened cheese	[49]
	*Penicillium* section	*Penicillium antarcticum*	Hard or semi-hard cheese	[49]
	*Canescentia*	*Penicillium arenicola*	Buffalo, goat, or sheep cheese	[56]
		*Penicillium canescens*	Hard or semi-hard cheese	[68]
		*Penicillium novae-zeelandiae*	Hard or semi-hard cheese	[54]
	*Penicillium* section *Charlesii*	*Penicillium charlesii*	Fresh unripened cheese	[49]
			Heat-treated milk	[49]
		*Penicillium dierckxii*	Fresh unripened cheese	[49]
	*Penicillium* section *Chrysogena*	*Penicillium chrysogenum*	Butter and margarine	[2]
			Blue-veined cheese	[63]
			Hard or semi-hard cheese	[54,59,62,65,69,70]
			Semi-soft cheese	[59]
			Buffalo, goat, or sheep cheese	[56]
			Fresh unripened cheese	[2]
			Yoghurt	[2]
		*Penicillium griseofulvum*	Hard or semi-hard cheese	[68]
		*Penicillium nalgiovense*	Hard or semi-hard cheese	[4,49,59]
			Semi-soft cheese	[4,59]
			Buffalo, goat, or sheep cheese	[56]
	*Penicillium* section *Citrina*	*Penicillium steckii*	Hard or semi-hard cheese	[69]
	*Penicillium* section *Exilicaulis*	*Penicillium corylophitum*	Hard or semi-hard cheese	[65]
	*Penicillium* section *Fasciculata*	*Penicillium aurantiogriseum*	Hard or semi-hard cheese	[65,69]
			Buffalo, goat, or sheep cheese	[56]
			Yoghurt	[2]
		*Penicillium camembertii*	Hard or semi-hard cheese	[68]
		*Penicillium commune*	Other dairy products	[49]
			Hard or semi-hard cheese	[2,4,49,54,59,65,69,70]
			Mold ripened cheese	[2]
			Semi-soft cheese	[4,59]
			Buffalo, goat, or sheep cheese	[56]
			Fresh unripened cheese	[2]
		*Penicillium crustosum*	Buffalo, goat, or sheep cheese	[56]
			Hard or semi-hard cheese	[54,59,62,65,69,70]
			Semi-soft cheese	[59]
			Buffalo, goat, or sheep cheese	[56]
		*Penicillium discolor*	Hard or semi-hard cheese	[49,59]
			Semi-soft cheese	[59]
		*Penicillium echinulatum*	Hard or semi-hard cheese	[59,65,68,69]
			Semi-soft cheese	[59]
		*Penicillium hirsutum*	Buffalo, goat, or sheep cheese	[56]
		*Penicillium nordicum*	Hard or semi-hard cheese	[49]
		*Penicillium palitans*	Fresh unripened cheese	[49]
			Hard or semi-hard cheese	[49,65,69]
		*Penicillium solitum*	Buffalo, goat, or sheep cheese	[56]
			Hard or semi-hard cheese	[2,49,54,59,65,69]
			Other dairy products	[49]
			Semi-soft cheese	[4,59]
			Yoghurt	[49]
		*Penicillium verrucosum*	Buffalo, goat, or sheep cheese	[56]
			Hard or semi-hard cheese	[54,59,68,69]
			Mold ripened cheese	[2]
			Semi-soft cheese	[4,59]
		*Penicillium viridicatum*	Hard or semi-hard cheese	[2,54,65,67,68,69]
	*Penicillium* section *Paradoxa*	*Penicillium atramentosum*	Blue-veined cheese	[63]
			Hard or semi-hard cheese	[59]
			Semi-soft cheese	[59]
	*Penicillium* section *Penicillium*	*Penicillium expansum*	Butter and margarine	[2]
			Hard or semi-hard cheese	[2,65,68,69]
	*Penicillium* section *Lanata-*	*Penicillium oxalicum*	Buffalo, goat, or sheep cheese	[56]
	*Divaricata*	*Penicillium simplicissimum*	Hard or semi-hard cheese	[57]
	*Penicillium* section	*Penicillium roquefortii*	Buffalo, goat, or sheep cheese	[56]
	*Roquefortum*		Hard or semi-hard cheese	[2,54,59,65,68,69]
			Mold ripened cheese	[2]
			Semi-soft cheese	[4,59]
	*Penicllium*	Nd *	Buffalo, goat, or sheep cheese	[56]
			Hard or semi-hard cheese	[54,66]
			Heat-treated milk	[57]
			Raw milk	[25,57]
	*Phaeramularia*	Nd *	Hard or semi-hard cheese	[54]
	*Phoma*	*Phoma glomerata*	Hard or semi-hard cheese	[2,70]
		*Phoma nebulosa*	Hard or semi-hard cheese	[69]
		Nd *	Buffalo, goat, or sheep cheese	[56]
			Hard or semi-hard cheese	[2,54,65,69]
	*Rhinocladiella*	Nd *	Blue-veined cheese	[63]
	*Sarocladium*	*Sarocladium strictum*	Hard or semi-hard cheese	[69]
	*Scopulariopsis*	*Scopulariopsis brevicaulis*	Buffalo, goat, or sheep cheese	[56]
			Hard or semi-hard cheese	[51,59]
			Semi-soft cheese	[4,59]
		Nd *	Hard or semi-hard cheese	[54,67]
	*Talaromyces*	*Talaromyces macrosporus*	Heat-treated milk	[2]
	*Trichoderma*	*Trichoderma harzianum*	Hard or semi-hard cheese	[65]
	*Ulocladium*	*Ulocladium chartarum*	Hard or semi-hard cheese	[69]
Basidiomycota	*Wallemia*	*Wallemia sebi*	Raw milk	[25]
Zygomycota	*Mucor*	*Mucor circinelloides*	Hard or semi-hard cheese	[49,69]
			Yoghurt	[2]
		*Mucor hiemalis*	Buffalo, goat, or sheep cheese	[56]
			Hard or semi-hard cheese	[2,65,70]
			Yoghurt	[2]
		*Mucor plumbeus*	Hard or semi-hard cheese	[65,69]
		*Mucor racemosus*	Hard or semi-hard cheese	[49,59,65,68,69]
			Semi-soft cheese	[59]
			Yoghurt	[2]
	*Rhizopus*	*Rhizopus stolonifer*	Blue-veined cheese	[63]
			Buffalo, goat, or sheep cheese	[56]
	*Thamnidium*	*Thamnidium elegans*	Hard or semi-hard cheese	[49]

* Nd: Not determined.

**Table 3:** List and regulations of chemical preservatives authorized in dairy products in the European Union (EU) [119] and the United States of America (USA) [120].

Preservatives	Dairy Product	USA Regulation	Food and Drug Administration (FDA) Code	EU Legislation	EU Code
Natamycin (pimaricin)	Cheese	20 mg/kg	172.155	-	-
	Uncut hard, semi-hard, and semi-soft cheese	20 mg/kg	172.155	1 mg/dm^2^, surface (not present at a depth of 5 mm)	E 235
Sorbic acid	Margarine	1000 mg/kg	182.3089	-	E 200
	Flavoured fermented milk	2000 mg/kg		1500 mg/kg	
	Non-heat-treated dairy-based desserts	2000 mg/kg		300 mg/kg	
	Whey cheeses, cheese products, processed cheeses	2000 mg/kg		2000 mg/kg	
	Curdled milk, unripened products, ripened products, pre-packed, sliced; layered ripened products	2000 mg/kg		1000 mg/kg	
Potassium sorbate and calcium sorbate	Cold-pack cheese, cream cheese, pasteurized process cheese food, pasteurized process cheese spread, semi-soft part-skim cheeses	3000 mg/kg	182.3640 and 182.3225	-	E 202 and E 203
	Flavoured fermented milk products including heat-treated products	3000 mg/kg		1500 mg/kg	
	Non-heat-treated dairy-based desserts	3000 mg/kg		300 mg/kg	
	Whey cheeses, cheese products, processed cheeses	3000 mg/kg		2000 mg/kg	
	Curdled milk, unripened products, ripened products, pre-packed, sliced; layered ripened products	3000 mg/kg		1000 mg/kg	
	Flavoured fermented milk products including heat-treated products	3000 mg/kg		1500 mg/kg	
	Non-heat-treated dairy-based desserts	3000 mg/kg		300 mg/kg	
	Whey cheeses, cheese products, processed cheeses	3000 mg/kg		2000 mg/kg	
	Curdled milk, unripened products, ripened products, pre-packed, sliced; layered ripened products	3000 mg/kg		1000 mg/kg	
Sodium benzoate	Margarine	1000 mg/kg	184.1733	-	E 211
	Flavoured fermented milk products including heat-treated products	-		1500 mg/kg	
	Non-heat-treated dairy-based desserts	-		300 mg/kg	
	Whey cheeses, cheese products, processed cheeses	-		2000 mg/kg	
	Curdled milk, unripened products, ripened products, pre-packed, sliced; layered ripened products	-		1000 mg/kg	
Potassium benzoate and calcium benzoate	Flavoured fermented milk products including heat-treated products	-	184.1081	1500 mg/kg	E 212 and E 213
	Non-heat-treated dairy-based desserts	-		300 mg/kg	
	Whey cheeses, cheese products, processed cheeses	-		2000 mg/kg	
	Curdled milk, unripened products, ripened products, pre-packed, sliced; layered ripened products	-		1000 mg/kg	
Propionic acid	Gruyere cheese, swiss cheese, and emmentaler cheese	surface		-	E 280
	Ripened cheese	-		surface treatment	
Sodium propionate	Cheeses and related cheese products	surface	184.1784	-	E 281
	Ripened cheese	-		surface treatment	

**Table 4:** Lactic and propionic acid bacteria showing antifungal activities and their responsible compounds.

Lactic Acid Bacteria (LAB) and Propionibacteria	Matrix	Metabolites	Targets	References
Genus *Lactococcus*				
*Lc. lactis*	Lab-Lemco tryptone broth (LTB)	Nd *	*Aspergillus flavus*	[162]
	LTB	Nisin	*Aspergillus parasiticus*	[163]
	Potatoe Dextrose Agar (PDA) + 0.1% Triton X-100	Nd *	*Aspergillus fumigatus*, *A. parasiticus*, *A. flavus*	[164]
	PDA + 0.1% Triton X-100	Possibly proteinaceous compound(s)	*Fusarium* spp., *A. flavus*, *A. parasiticus*	[165]
	LTB and PDA	Possibly proteinaceous compound(s), lactic acid	*Penicillium expansum*	[166]
Genus *Lactobacillus*				
*Lactobacillus* spp.	De Man, Rogosa and Sharpe (MRS) agar	Acetic acid, propionic acid, lactic acid, peptides	*Penicillium candidum*, *Debaryomyces hansenii*	[167]
Group *Lb. alimentarius/Lb. farciminis*				
*Lb. paralimentarus*	Modified Sabouraud Dextrose Broth (mSDB) medium	Lactic acid, phenyllactic acid, acetic acid, peptides	*Aspergillus japonicus, Eurotium repens, Penicillium roseopurpureum*	[168]
Group *Lb. brevis*				
*Lb. brevis*	PDA	Peptide	*Penicillium roqueforti*	[169]
	wheat flour hydrolysate (WFH) broth	Acetic acid, phenyllactic acid, lactic acid	*Fusarium graminearum*	[170]
	mMRS agar	Organic acids and proteinaceous compounds	*Fusarium* spp.	[171]
	MRS agar or PDA	Peptide	*Penicillium camemberti, P. roqueforti, Aspergillus niger, Rhizopus oryzae, Kluyveromyces marxianus, Torulopsis candida, Meyerozyma guillermondii, Saccharomyces cerevisiae*	[172]
Group *Lb. casei*				
*Lb. casei*	PDA + 0.1% Triton X-100	Possibly proteinaceous compound(s)	*A. flavus, A. parasiticus, Fusarium* sp.	[165]
	PDA	Peptide	*Penicillium citrinum, P. expansum, A. flavus*	[173]
	Yoghurt	Lactic acid and cyclo-(Leu-Pro)	*Penicillium* sp.	[174]
	LTB and PDA	Possibly proteinaceous compound(s), lactic acid	*P. expansum*	[166]
*Lb. paracasei*	Yoghurt and cheese surface	Propionic acid, acetic acid, lactic acid, succinic acid, 2-pyrrolidone-5-carboxylic acid, 3-phynyllactic acid, hydroxyphenyllactic acid	*Candida pulcherrima*, *Candida magnoliae*, *Candida parapsilosis*, *Zygosaccharomyces bailii*	[151]
	MRS agar	Peptide	*Candida albicans, Candida blankii, Candida pseudointermedia*	[175]
	Chemically defined interaction medium	Diacetyl	*Penicillium solitum, Penicillium* sp.	[153]
	Yoghurt and acidified milk	Diacetyl, acetic acid, butanoic acid, 2,3-pentadione	*P. solitum, Penicillium* sp.	[153]
*Lb. rhamnosus*	Yoghurt	Acetic acid, lactic acid	*Rhodotorula mucilaginosa*	[15,158]
Group *Lb. coryniformis*				
*Lb. coryniformis*	MRS agar or PDA	Peptide +/−3 KDa, phenyllactic acid, cyclo(Phe-Pro), cyclo(Phe-OH-Pro), reuterin	*Broad spectrum*	[147]
Group *Lb. delbrueckii*				
*Lb. acidophilus*	PDA + 0.1% Triton X	Nd *	*A. fumigatus*	[164]
*Lb. amylovorus*	MRS broth	3-phenylpropanoic acid, *p*-coumaric, (E)-2-methylcinnamic acid, 3-phenyllactic acid, 3-(4-hydroxyphenyl)lactic acid, lactic acid, acetic acid, d-glucuronic acid, salicylic acid, cytidine and 2′-deoxycytidine, sodium decanoate,	*A. fumigatus, Fusarium culmorum*	[175]
	Cheese	Cyclo(l-Pro-l-Pro), cyclo(l-Leu-l-Pro), cyclo(l-Tyr-l-Pro) cyclo(l-Met-l-Pro) and cyclo(l-His-l-Pro)	*P. roqueforti, P. expansum*	[176]
	Milk agar and cheese	dl-ρ-hydroxyphenyllactic acid, 4-hydroxybenzoic acid, (S)-(−)-2-hydroxyisocapric acid, azelaic acid, phenyllactic acid, benzoic acid, hydrocinnacmic acid, 3-hydroxydecanoic acid, dl-β-hydroxylauric acid, decanoic acid, salicylic acid, 4-hydroxybenzoic, vanillic acid, (*S*)-(−)-2-hydroxyisocapric acid	*P. expansum*	[177]
*Lb. delbrueckii*	LTB and PDA	Possibly proteinaceous compound(s), lactic acid	*P. expansum*	[166]
Group *Lb. fructivorans*				
*Lb. sanfranciscencis*	Malt-agar medium	Caproic acid, propionic acid, butyrix acid, acetic acid, valeric acid	*F. graminearum*	[178,179]
Group *Lb. perolens*				
*Lb. harbinensis*	Yoghurt	Acetic acid, lactic acid	*Yarrowia lipolytica, P. expansum, Penicillium brevicompactum, D. hansenii, R. mucilaginosa, Kluyveromyces lactis*	[15,158]
Group *Lb. plantarum*				
*Lb. pentosus*	MRS	Peptide	*A. niger*	[180]
	MRS agar	Peptide, phenyllactic and hydroxyphenyllactic acid	*Penicillium nalgiovense, Aspergillus candidus*	[181]
*Lb. plantarum*	mMRS agar	(*S*)-(−)-2-hydroxyisocapric acid, hydrocinnamic acid, phenyllactic acid, decanoic acid, azelaic acid, 4-hydroxybenzoic acid, *p*-coumaric acid, vanillic acid, dl-Þ-hydroxyphenyllactic acid, 3-hydroxydecanoic acid	*Microsporum canis, Microsporum gypseum, Epidermophyton floccosum*	[182]
	MRS broth	Benzoic acid, 5-methyl-2,4-imidazolidinedione, tetrahydro-4-hydroxy-4-methyl-2H-pyran-2-one, 3-(2-methylpropyl)-2,5-piperazinedione, cyclo(glycyl-l-leucyl)).	*Fusarium avenaceum*	[183]
	MRS agar	Lactic acid, PLA, cyclo(l-Leu-l-Pro), cyclo(l-Phe-l-Pro)	*A. niger, F. graminearum, F. culmorum, Fusarium oxysporum*	[184]
	Wheat flour hydrolysate (WFH)	Phenyllactic acid, 4-hydroxy-phenillactic acid	*E. repens, Eurotium rubrum, Penicillium corylophilum, P. roqueforti, P. expansum, Endomyces fibuliger, A. niger, A. flavus, Monilia sitophila, F. graminearum*	[185]
	MRS broth	3-phenyllactic acid, cyclo(Phe-Pro), cyclo(Phe-OH-Pro), cyclo(l-Phe-LPro) and cyclo(l-Phe-*trans*-4-OH-l-Pro) dipeptides	*Fusarium sporotrichioides, A. fumigatus, K. marxianus*	[186]
	Wheat flour hydrolysate (WFH)	Acetic acid, phenyllactic acid, lactic acid	*F. graminearum, A. niger*	[170]
	MRS agar	3-(*R*)-hydroxydecanoic acid, 3-hydroxy-5-cis-dodecenoic acid, 3-(*R*)-hydroxydodecanoic acid and 3-(*R*)-hydroxytetradecanoic acid	*A. fumigatus, Aspergillus nidulans, K. marxianus, P. roqueforti, Penicillium commune, Penicillium anomala, R. mucilaginosa*	[187]
	MRS agar plates	Acetic acid	*A. flavus, F. graminearum, Rhizopus stolonifer, Sclerotium oryzae, Rhizoctonia solani, Botrytis cinerea, Sclerotinia minor*	[188]
	MRS agar	Peptide, phenyl-lactic and hydroxy-phenyllactic acid	*P. nalgiovense, P. camemberti, Penicillium verrucosum, Penicillium chrysogenum, A. candidus, A. flavus, A. ochraceus, A. fumigatus, Galactomyces geotrichum, Moniliella* spp., *Mucor racemosus, Wallemia sebi, Eurotium herbariorum*	[181]
	Soybean	3,6-bis(2-methylpropyl)-2,5-piperazinedion	*A. flavus*	[189]
	MRS agar plates	3-3-phenyllactic acid (PLA), lactic acid, acetic acid	*A. fumigatus, Rhizopus stolonifer*	[190]
	MRS agar medium, apple-based agar growth medium	Lactic acid, acetic acid	*P. expansum, Penicillium notatum*	[191]
	MRS agar medium	2-hydroxy-4-methylpentanoic acid	*A. niger, Aspergillus tubingensis, Penicillium crustosum*	[192]
	PDA plates	3-PLA, benzeneacetic acid, 2-propenyl ester	*B. cinerea, Glomerella cingulate, Phytophthora drechsleri Tucker, P. citrinum, Penicillium digitatum, F. oxysporum*	[193]
	Chopped Meat Carbohydrate (CMC) broth	Lactic acid	*F. avenaceum, F. culmorum, F. graminearum, F. oxyporum*	[194]
Group *Lb. reuteri*				
*Lb. fermentum*	MRS	Peptide	*A. niger*	[180]
*Lb. reuteri*	Wheat flour hydrolysate (WFH) broth	Acetic acid, phenyllactic acid, lactic acid	*F. graminearum, A. niger*	[170]
	mMRS agar	Acid, vanillic acid, dl-Þ-hydroxyphenyllactic acid, 3-hydroxydecanoic acid, (*S*)-(-)-2–hydroxyisocapric acid, hydrocinnamic acid, phenyllactic acid, decanoic acid, azelaic acid, 4-hydroxybenzoic acid, p-coumaric	*M. canis, M. gypseum, E. floccosum*	[182]
*Lb. rossiae*	mSDB agar medium	Lactic acid, phenyllactic acid, acetic acid, peptides	*A. japonicus, E. repens, P. roseopurpureum*	[168]
Group *Lb. sakei*				
*Lb. sakei*	MRS	Sakacin KTU05-6	*A. flavus, A. fumigatus, A. niger, Aspergillus versicolor, F. culmorum, Fusarium poae, Mucor* spp., *P. chrysogenum, P. expansum, Penicillium* spp.	[195]
Genus *Pediococcus*				
*Pc. acidilactici*	MRS	Pediocin KTU05-08	*A. flavus, A. fumigatus, A. niger, A. versicolor, F. culmorum, F. poae, Mucor* spp., *P. chrysogenum, P. expansum, Penicillium* spp.	[195]
*Pc. pentosaceous*	MRS	Peptide	*A. niger*	[180]
	MRS agar plates	Acetic acid	*A. flavus, F. graminearum, R. stolonifer, S. oryzae, R. solani, B. cinerea, S. minor*	[188]
	MRS	Pediocin KTU05-09, KTU05-10 and AcKTU05-67	*A. flavus, A. fumigatus, A. niger, A. versicolor, F. culmorum, F. poae, Mucor* spp., *P. chrysogenum, P. expansum, Penicillium* spp.	[195]
	MRS agar medium	Possibly cyclic dipeptide	*P. expansum, Penicillium notatum*	[191]
Genus *Weissella*				
*W. confusa*	MRS agar medium	Lactic acid, acetic acid	*P. expansum, P. notatum*	[191]
*W. cibaria*	MRS agar medium	Lactic acid, acetic acid	*P. expansum, P. notatum*	[191]
*W. paramesenteroides*	MRS agar medium	2-hydroxy-4-methylpentanoic acid, lactic acid, acetic acid	*A. niger, A. tubingensis, P. crustosum*	[192]
	MRS agar plates	Acetic acid	*A. flavus, F. graminearum, R. stolonifer, S. oryzae, B. cinerea, S. minor*	[188]
Genus *Propionibacteria*				
*P. acidipropionici*	Sodium lactate (SL) medium	Lactic acid, propionic acid, acetic acid	*A. fumigatus*	[196]
	MRS	Propionic acid, acetic acid	*R. mucilaginosa, P. roqueforti, A. fumigatus*	[196]
	MRS-acetate	Propionic acid, acetic acid	*R. mucilaginosa, P. roqueforti, A. fumigatus*	[196]
	SL broth	PLA	*A. fumigatus*	[196]
*P. freudenreichii* subsp. *shermanii*	Skim milk	propionic acid, 3-phenyllactic acid	*P. chrysogenum*	[159]
	MRS	Acetic acid, propionic acid, 3-phenyllactic acid, 4-hydroxy-phenyllactic acid	*P. chrysogenum*	[159]
	MRS	Propionic acid, acetic acid	*P. roqueforti, A. fumigatus*	[196]
	MRS-acetate	Propionic acid, acetic acid	*R. mucilaginosa, P. roqueforti, A. fumigatus*	[196]
*P. freudenreichii* subsp. *freudenreichii*	MRS	Propionic acid, acetic acid	*R. mucilaginosa, P. roqueforti, A. fumigatus*	[196]
	MRS-acetate	Propionic acid, acetic acid	*R. mucilaginosa, P. roqueforti, A. fumigatus*	[196]
*P. jensenii*	Yoghurt and cheese surface	Propionic acid, acetic acid, lactic acid, succinic acid, 2-pyrrolidone-5-carboxylic acid, 3-phynyllactic acid, hydroxyphenyllactic acid	*C. pulcherrima, C. magnollae, C. parapsilosis, Z. bailii*	[151]
	MRS	Propionic acid, acetic acid	*R. mucilaginosa, P. roqueforti, A. fumigatus*	[196]
	MRS-acetate	Nd *	*R. mucilaginosa, P. roqueforti, A. fumigatus*	[196]
*P. thoenii*	Sodium Lactate (SL) medium	Lactic acid, propionic acid	*A. fumigatus*	[196]
	SL broth	PLA	*A. fumigatus*	[196]
	MRS	Propionic acid, acetic acid	*R. mucilaginosa, P. roqueforti, A. fumigatus, K. marxianus*	[196]
	MRS-acetate	Propionic acid, acetic acid	*R. mucilaginosa, P. roqueforti, A. fumigatus*	[196]

* Nd: not determined.
